# Amorphous nano-selenium quantum dots prevent pulmonary arterial hypertension through recoupling endothelial nitric oxide synthase

**DOI:** 10.18632/aging.202215

**Published:** 2020-12-15

**Authors:** Mo-Li Zhu, Zhi-Tao Gao, Jun-Xiu Lu, Yang Wang, Ge Wang, Tian-Tian Zhu, Peng Li, Chao Liu, Shuang-Xi Wang, Lin Yang

**Affiliations:** 1 Collaborative Innovation Center of Henan Province for Green Manufacturing of Fine Chemicals, Key Laboratory of Green Chemical Media and Reactions, Ministry of Education, School of Chemistry and Chemical Engineering, Henan Normal University, Xinxiang, Henan, China; 2 Henan International Joint Laboratory of Cardiovascular Remodeling and Drug Intervention, School of Pharmacy, Xinxiang Medical University, Xinxiang, Henan, China; 3 School of Laboratory Medicine, Xinxiang Medical University, Xinxiang, Henan, China; 4 School of Basic Medical Sciences, Xinxiang Medical University, Xinxiang, Henan, China; 5 Department of Pharmacy, The 3rd Affiliated Hospital of Xinxiang Medical University, Xinxiang, Henan, China; 6 Hubei Key Laboratory of Diabetes and Angiopathy, Hubei University of Science and Technology, Xianning, Hubei, China; 7 The Key Laboratory of Cardiovascular Remodeling and Function Research, Chinese Ministry of Education, Chinese National Health Commission and Chinese Academy of Medical Sciences, The State and Shandong Province Joint Key Laboratory of Translational Cardiovascular Medicine, Qilu Hospital of Shandong University, Jinan, Shandong, China

**Keywords:** nano-selenium, pulmonary arterial hypertension, endothelial nitric oxide synthase, tetrahydrobiopterin

## Abstract

Aims: We have previously reported that nano-selenium quantum dots (SeQDs) prevented endothelial dysfunction in atherosclerosis. This study is to investigate whether amorphous SeQDs (A-SeQDs) increase endogenous tetrahydrobiopterin biosynthesis to alleviate pulmonary arterial hypertension.

Results: Both A-SeQDs and C-SeQDs were stable under physiological conditions, while the size of A-SeQDs was smaller than C-SeQDs by high resolution-transmission electron microscopy scanning. In monocrotaline-injected mice, oral administration of A-SeQDs was more effective to decrease pulmonary arterial pressure, compared to C-SeQDs and organic selenium. Further, A-SeQDs increased both nitric oxide productions and intracellular BH4 levels, upregulated dihydrofolate reductase activity in lungs, and improved pulmonary arterial remodeling. Gene deletion of dihydrofolate reductase abolished these effects produced by A-SeQDs in mice. Finally, the blood levels of tetrahydrobiopterin and selenium were decreased in patients with pulmonary arterial hypertension.

Conclusion: A-SeQDs increase intracellular tetrahydrobiopterin to prevent pulmonary arterial hypertension through recoupling endothelial nitric oxide synthase.

Methods: Two polymorphs of SeQDs and A-SeQDs, and a crystalline form of SeQDs (C-SeQDs) were prepared through self-redox decomposition of selenosulfate precursor. Mice were injected with monocrotaline to induce pulmonary arterial hypertension *in vivo*. Pulmonary arterial pressure was measured.

## INTRODUCTION

Pulmonary arterial hypertension (PAH) is a serious disease which is highlighted by pulmonary hypervasoconstriction [1]. Despite the high mortality, no efficient treatments have been established. The endothelial nitric oxide (NO) synthase (eNOS) plays a critical role in maintaining normal pulmonary vascular tension. Uncoupled eNOS is able to imbalance NO and reactive oxygen species (ROS) productions in the pulmonary artery, resulting in the pathogenesis of PAH.

Tetrahydrobiopterin (BH4) is required for eNOS coupling to produce NO or ROS [2]. Mice deficient in the rate-limiting enzyme of BH4 biosynthesis, such as GTP cyclohydrolase 1 and dihydrofolate reductase (DHFR), develop the phenotype of PAH under normal conditions [3–5]. It has been reported that BH4 supplementation therapy by L-sepiapterin recouples eNOS and ameliorates hypoxia-induced PAH in newborn pigs [6]. However, it is not suitable for chronic oral administration because BH4 is highly oxidized. A pharmacological approach that can effectively elevate intracellular BH4 level is currently lacking.

Selenium is a vital component of the selenium-dependent proteins that have several biological functions in human health [7]. Although it is very important for human health, selenium is not utilized as a free element in the human body. Selenium is relatively rare in nature, and so, it is very unevenly distributed in each organ of the body. Selenium is rich in the livers and kidneys, while it is very poor in the lungs [8]. Low level of serum selenium might cause lung cancer and affect the development of neonatal rat lungs [9, 10].

Nanomaterials have been widely used in nano-imaging, therapeutics, etc. Nano-selenium quantum dots (SeQDs) are expected to be a new therapeutic drug because of the unique and chemical physical properties [11, 12]. Though AK. Zamani Moghaddam et al improved the approach of selenium supplementation by nano-selenium to treat Broiler Chickens with PAH, the effects were minor and only to reverse right ventricle hypertrophy [13]. In this study, we made amorphous SeQDs (A-SeQDs) and crystalline SeQDs (C-SeQDs), and observed that A-SeQDs displayed more excellent effects on monocrotaline-induced PAH than C-SeQDs or organic selenium. Mechanically, A-SeQDs, rather than C-SeQDs and organic selenium, were able to distribute into lung to activate DHFR. In pulmonary arterial endothelial cells, A-SeQDs increased DHFR-dependent BH4 salvage pathway.

## RESULTS

### The morphology of A-SeQDs was different to C-SeQDs

**Figure 1:**
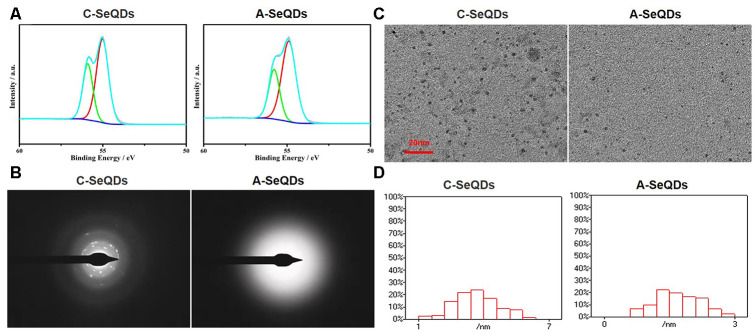
**Basic morphological characterizations of A-SeQDs and C-SeQDs.** (**A**) The compositions of A-SeQDs and C-SeQDs were determined by the analysis of X-ray photoelectron spectroscopy. (**B**) The halo rings in A-SeQDs and C-SeQDs were observed by using electron diffraction pattern in the selected-area. (**C**) The diameters of A-SeQDs and C-SeQDs were observed by high resolution-transmission electron microscope. (**D**) The diameter’s distributions of both A-SeQDs and C-SeQDs were shown.

We firstly generated two polymorphs of SeQDs through self-redox decomposition of selenosulfate precursor in the presence of BSA and measured the chemical compositions of two SeQDs by using X-ray photoelectron spectroscopy. As indicated in Figure 1A, the binding energy of either C-SeQDs or A-SeQDs was approximately 55 eV, suggesting both C-SeQDs and A-SeQDs consist of selenium element but not compound. While, X-ray diffraction pattern analysis revealed that the sample of A-SeQDs prepared at 25° C for 12 hours had no obvious diffraction peaks, while the sample of C-SeQDs prepared at 80° C for 36 hours revealed multiple diffraction peaks (Supplementary Figure 1C). Further, by using the analysis of the selected-area electron diffraction pattern (Figure 1B), A-SeQDs revealed a diffused halo ring, rather than any detectable rings or spots, indicating the formation of the amorphous product. Reversely, C-SeQDs exhibits the obvious diffraction spots, confirming the formation of the crystalline product. These data suggest that the morphology of A-SeQDs was amorphous but not crystalline, though both A-SeQDs and C-SeQDs are selenium element.

### The size of A-SeQDs was smaller than C-SeQDs

We next examined the sizes of A-SeQDs and C-SeQDs by using high resolution-transmission electron microscopy. As shown in Figure 1C, the size of A-SeQDs was smaller than C-SeQDs (2.25±0.19 *VS* 4.10±0.04 nm, *P*<0.05). By calculating the frequency of SeQDs sizes, the diameter of C-SeQDs was in normal distribution, but the diameter of A-SeQDs was in abnormal distribution (Figure 1D).

### Both A-SeQDs and C-SeQDs were stable in different solutions

The stabilities of A-SeQDs and C-SeQDs in different vehicles, such as ddH_2_O, PBS, and DMEM, were examined by performing ξ-potential measurement (Supplementary Figure 2 and Supplementary Table 1), indicating that both A-SeQDs and C-SeQDs were stable in solutions.

### Administration of organ selenium supplementation or C-SeQDs partially prevented the formation of PAH in monocrotaline-injected mice

**Figure 2:**
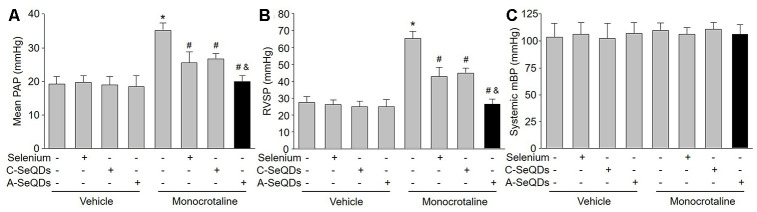
**Administration of A-SeQDs is more effective to prevent monocrotaline-induced PAH than C-SeQDs and selenium supplementation in mice.** The experimental protocol was shown in Supplementary Figure 3A. C57B16 mice were given selenium supplementation, C-SeQDs, A-SeQDs in regular diet one week prior to a single intraperitoneal injection of 100 mg/kg monocrotaline or vehicle. Three weeks after injection, mean pulmonary arterial pressure (PAP) in (**A**), right ventricle systolic pressure (RVSP) in (**B**), and mean blood pressure (mBP) in (**C**) were measured by radio telemetry. All data were expressed as mean ± SEM. 10-15 mice were in each group. *P<0.05 *VS* Vehicle alone, ^#^*P* < 0.05 *VS* monocrotaline alone. ^&^*P*<0.05 *VS* monocrotaline plus Selenium or C-SeQDs. A one-way ANOVA followed by Tukey *post-hoc* tests was used to produce the P values.

We then generated the PAH model by injecting monocrotaline into mice to investigate the effects of organ selenium, A-SeQDs or C-SeQDs on PAH (Supplementary Figure 3A). As indicated in Figure 2A and 2B, after 3 weeks’ injection of monocrotaline, PAP was increased from 18.3±0.2 mmHg to 34.7±0.3 mmHg. RVSP was also increased from 27.2±0.3 mmHg to 65.2±0.5 mmHg, demonstrating the PAH model was successfully established in mice. Treatments with either organic selenium supplementation or C-SeQDs in monocrotaline-injected mice partially reduced RVSP and PAP. Systemic mean blood pressure was not affected by monocrotaline injection, selenium supplementation or C-SeQDs administration (Figure 2C).

### A-SeQDs were more effective to prevent PAH than selenium supplementation and C-SeQDs in mice

We thought A-SeQDs might be more effective to improve PAH since the diameters of A-SeQDs were much smaller than C-SeQDs. Therefore, we compared the effects of A-SeQDs to selenium supplementation and C-SeQDs. As expected, A-SeQDs did not only reduce both PAP and RVSP in monocrotaline-injected mice, but it was much stronger than either selenium supplementation or C-SeQDs to improve PAH in mice with PAH (Figure 2A and 2B).

### A-SeQDs improved right ventricle hypertrophy in hearts isolated from monocrotaline-injected mice

**Figure 3:**
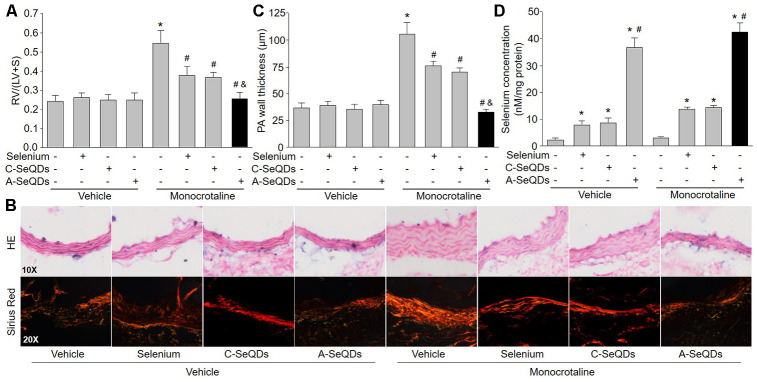
**Administration of A-SeQDs improves pulmonary arterial remodeling in monocrotaline-injected mice.** The experimental protocol was shown in Supplementary Figure 3A. At the end of experiment, mice were sacrificed under anesthesia. (**A**) The hearts were isolated to measure the ratio of right ventricle (RV) to left ventricle (LV) plus septum (S) weights [RV/(LV+S)]. (**B** and **C**) Pulmonary arteries were subjected to perform HE staining and sirius red staining in B. The thickness of pulmonary artery was calculated in C. All data were expressed as mean ± SEM. 10-15 mice were in each group. *P<0.05 *VS* Vehicle alone, ^#^*P*<0.05 *VS* monocrotaline alone. ^&^*P*<0.05 *VS* monocrotaline plus Selenium or C-SeQDs. (**D**) The content of selenium in lung tissue was determined. *P<0.05 *VS* Vehicle or monocrotaline alone, ^#^*P*<0.05 *VS*. monocrotaline plus Selenium or C-SeQDs. A one-way ANOVA followed by Tukey *post-hoc* tests was used to produce the P values.

One of the PAH outcomes is right ventricle hypertrophy [14]. We next checked the right ventricle remodeling in monocrotaline-injected mice. As shown in Figure 3A–3C, three kinds of selenium therapy improved right ventricle remodeling by reducing RV/(LV+S) the thickness and collagen depositions in pulmonary arterial walls in monocrotaline-injected mice. However, the inhibitions of right ventricle hypertrophy induced by A-SeQDs were much more obvious than either organic selenium supplementation or C-SeQDs in mice with PAH. Collectively, these data suggest that A-SeQDs were more effective to prevent PAH than organic selenium and crystalline selenium.

### A-SeQDs increased the levels of selenium in lungs in mice

In normal condition, selenium is rarely distributed in lung [10]. We next examined if three kinds of selenium therapy elevated selenium concentration in lung tissue. Interestingly, the selenium concentration in lungs isolated from mice treated with A-SeQDs was dramatically increased (Figure 3D). Organic selenium supplementation and C-SeQDs slightly increased selenium levels in lung tissue. Their effects were much weaker than A-SeQDs. This may explain why A-SeQDs produce much stronger effects on PAH than organic selenium and C-SeQDs.

### A-SeQDs increased NO production and eNOS activity, but decreased ROS levels in pulmonary arteries isolated from mice with PAH

**Figure 4:**
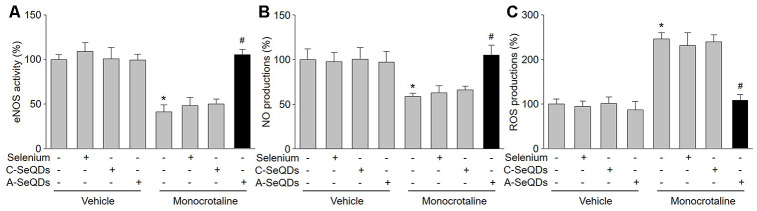
**A-SeQDs recouple eNOS in pulmonary arteries of monocrotaline-injected mice**. The experimental protocol was shown in Supplementary Figure A. At the end of experiment, mice were sacrificed under anesthesia. Pulmonary artery isolated from mice were subjected to measure NO productions in (**A**), ROS productions in (**B**), and eNOS activity in (**C**). All data were expressed as mean ± SEM. 10-15 mice were in each group. *P<0.05 *VS* Vehicle alone, ^#^*P* < 0.05 *VS* monocrotaline alone. A one-way ANOVA followed by Tukey *post-hoc* tests was used to produce the P values.

PAH in early stage is characterized by endothelial dysfunction due to eNOS uncoupling, in which eNOS produces ROS but not NO, leading to pulmonary arterial vasoconstriction [15]. Therefore, we examined the effects of A-SeQDs on eNOS uncoupling by measuring NO production, ROS generation, and eNOS activity in pulmonary arterial walls. As represented in Figure 4A–4C, injection of monocrotaline decreased eNOS activity and NO production, but increased ROS generation in pulmonary artery. As a sequence, these detrimental effects induced by monocrotaline were bypassed by A-SeQDs.

### A-SeQDs increased BH4 salvage pathway in monocrotaline-injected mice

**Figure 5:**
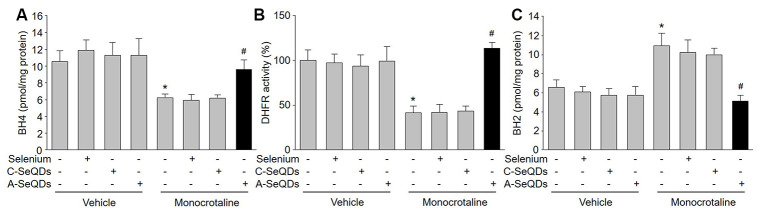
**Administration of A-SeQDs activates BH4 salvage pathway in pulmonary arteries of monocrotaline-injected mice.** The experimental protocol was shown in Supplementary Figure 3A. At the end of experiment, mice were sacrificed under anesthesia. Pulmonary artery isolated from mice were subjected to measure BH4 in (**A**), DHFR activity in (**B**), and BH2 in (**C**). All data were expressed as mean ± SEM. 10-15 mice were in each group. *P<0.05 *VS* Vehicle alone, ^#^*P* < 0.05 *VS* monocrotaline alone. A one-way ANOVA followed by Tukey *post-hoc* tests was used to produce the P values.

BH4 is a critical factor for determining eNOS to produce NO or ROS in endothelial cells [16]. Intracellular BH4 levels may be influenced by oxidation, forming BH2. DHFR can regenerate BH4 from BH2 via salvage pathway. As indicated in Figure 5A and 5B, both BH4 concentration and DHFR activity were increased by A-SeQDs in monocrotaline-injected mice. Reversely, BH2 concentration was decreased in monocrotaline-injected mice if treated with A-SeQDs (Figure 5C). However, DHFR protein levels were not affected by organic selenium, C-SeQDs or A-SeQDs (Supplementary Figure 3B). These data indicate that A-SeQDs prevent PAH, which is possibly associated with activation of BH4 salvage pathway.

### A-SeQDs-induced alleviation of PAH is DHFR dependent in mice

**Figure 6:**
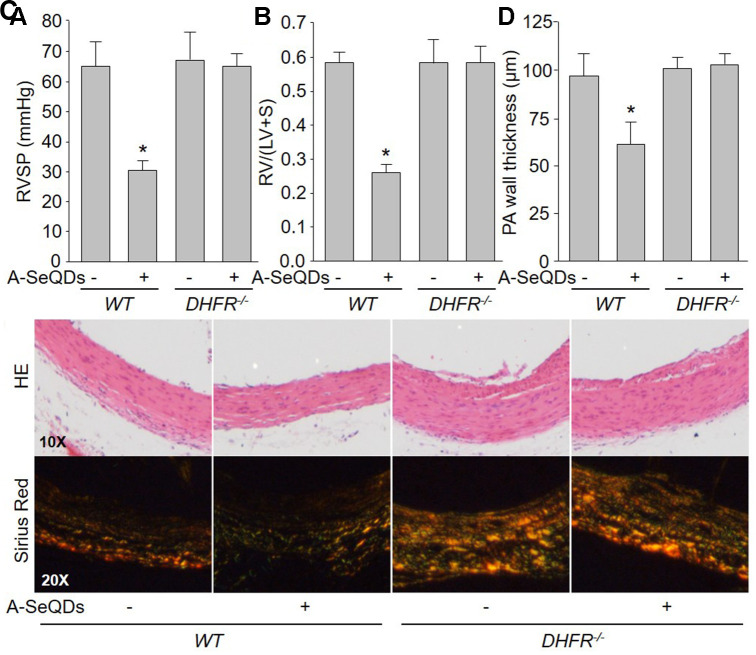
**A-SeQDs-induced alleviation of PAH is DHFR dependent in monocrotaline-injected mice.** The experimental protocol was shown in Supplementary Figure 3A. Wildtype *(WT*) and *DHFR^-/-^* mice were given A-SeQDs administration in regular diet one week prior to a single intraperitoneal injection of 100 mg/kg monocrotaline. Three weeks after injection, right ventricle systolic pressure (RVSP) in (**A**) and the ratio of right ventricle (RV) to left ventricle (LV) plus septum (S) weights [RV/(LV+S)] in (**B**) were measured. Pulmonary arteries isolated from mice were subjected to perform HE staining and sirius red staining in (**C**) and the thickness of pulmonary artery was calculated in (**D**). All data were expressed as mean ± SEM. 10-15 mice were in each group. *P<0.05 *VS* *WT* alone. A one-way ANOVA followed by Tukey *post-hoc* tests was used to produce the P values.

To determine the role of DHFR in the improvement of PAH induced by A-SeQDs, we compared the effects of A-SeQDs on PAH in *WT* and *DHFR^-/-^* mice (Supplementary Figure 3C). As shown in Figure 6A–6D, though A-SeQDs prevented the formation of PAH in *WT* mice injected with monocrotaline, it did not reduce RVSP, RV/(LV+S), the thickness and collagen depositions in pulmonary arterial walls in *DHFR^-/-^* mice, suggesting that these beneficial effects of A-SeQDs on PAH depend on DHFR.

### Gene deletion of DHFR abolishes the effects of A-SeQDs on BH4 salvage synthesis and eNOS recoupling in mice

**Figure 7:**
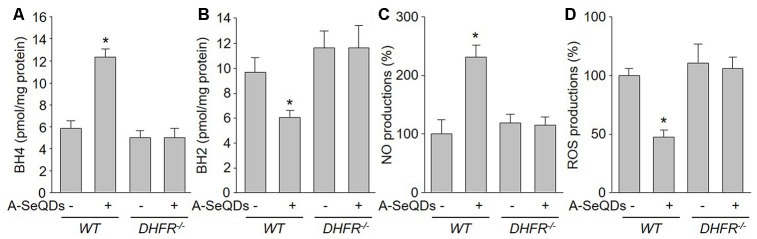
**Gene deletion of DHFR abolishes the effects of A-SeQDs on BH4 salvage synthesis and eNOS recoupling in pulmonary arteries isolated from monocrotaline-injected mice.** The experimental protocol was shown in Supplementary Figure 3A. Three weeks after monocrotaline injection, pulmonary artery isolated from mice were subject to measure BH4 in (**A**), BH2 in (**B**), NO productions in (**C**), and ROS productions in (**D**). All data were expressed as mean ± SEM. 10-15 mice were in each group. *P<0.05 *VS* *WT* alone. A one-way ANOVA followed by Tukey *post-hoc* tests was used to produce the P values.

Since we have identified the crucial role of DHFR in A-SeQDs-suppressed PAH formation, we next determined whether DHFR mediates the effects of A-SeQDs on BH4 salvage pathway and eNOS recoupling. As illustrated in Figure 7A–7D, A-SeQDs increased BH4 content and NO production, and decreased BH2 level and ROS generation in pulmonary arterial walls isolated from monocrotaline-injected *WT* mice, but not reversed these abnormalities in monocrotaline-injected *DHFR^-/-^* mice. These data indicate that A-SeQDs-induced eNOS recoupling is mediated by DHFR-dependent activation of BH4 salvage pathway.

### Reduced levels of BH4, selenium, and DHFR activity in human subjects with PAH

In order to establish the clinical association between selenium and PAH in human subjects, we determined the level of serum selenium in patients with PAH. We conducted a pilot experiment by collecting samples of blood from human subjects with or without PAH. The demographic data of human subjects were presented in Supplementary Table 2. As shown in Supplementary Figure 4A and 4B, the levels of BH4 and DHFR activity were significantly reduced in human subjects with PAH, compared to healthy humans. Importantly, serum selenium levels were also decreased in human subjects with PAH (Supplementary Figure 4C). Although the pilot experiment was unable to establish the cause-effect relation between selenium and PAH in clinical investigations, it still implies the importance of selenium supplementation through amorphous nanoparticles in human PAH patients.

## DISCUSSION

In this study, we reported that A-SeQDs rather than C-SeQDs are able to distribute to lung, resulting in DHFR-dependent upregulation of BH4 salvage synthesis in pulmonary arterial endothelial cells. Gene knockout of DHFR abolished A-SeQDs-increased intracellular BH4 levels and eNOS recoupling in mice. The present project is the first to indicate that administration of selenium therapy prevents PAH through the activation of BH4 salvage pathway.

A big discovery of the present study is that we firstly developed a novel approach to supply selenium through amorphous nanoparticles to prevent PAH. Though the therapy of selenium supplementation has been recognized can prevent multiple diseases if it is optimally absorbed in body. The selenium speciation is crucial to determine the effects of selenium supplementation [7]. The security dose of selenium in human body is very narrow, which limits the wide application of traditional selenium compounds. Thus, it is essential to develop an efficient and safe form of selenium. Further, selenium is hardly distributed into lung, causing the lack of effects by selenium therapy in lung. In this study, by generating selenium in the form of amorphous nano quantum dots, selenium is able to distribute into lung to produce beneficial effects on PAH. Our studies are in line with an earlier report that selenium supplementation by nano-selenium lowers PAH in Broiler Chickens, but the effects were minor and only to reverse right ventricle hypertrophy [13]. Why A-SeQDs, but not C-SeQDs and organic selenium, are able to penetrate lung tissue needs further observations.

The other discovery is that selenium is an activator of DHFR to increase BH4 salvage pathway in endothelial cells. Selenium, an essential trace element, plays an important role in human health [17]. Many studies shows that organic selenium might inhibit atherosclerosis in animals, in which it is involved in lowering serum lipids, reducing oxidative stress, modulating inflammation, and protecting vascular cells [7, 18, 19]. In this study, supplementation of organic selenium or C-SeQDs partially reversed the formation of PAH, we thought their effects on PAH were due to these actions, in which these effects are produced in the whole body but not in lung because they did not increase selenium concentration in lung. While, A-SeQDs are able to distribute into lung to activate DHFR to increase BH4 salvage pathway in pulmonary arterial endothelial cells. In this way, A-SeQDs recouple eNOS to lower PAP.

An issue remained is how selenium activates DHFR activity in endothelial cells. Selenium is incorporated into selenoproteins to prevent some diseases [20]. Selenium, in the methylated form, is a good choice for selenium supplementation to correct a deficiency. In the present study, by using nanomaterials, we observed that A-SeQDs activated DHFR in monocrotaline-injected mice. We reasoned multiple pathways, such as Na^+^/H^+^ exchanger 1, AMP-activated protein kinase, Akt, and prostacyclin synthase in endothelial cell, may mediate this biological action of A-SeQDs on DHFR activation because these mediators are involved in endothelial dysfunction in cardiovascular diseases [21–25]. What selenium-dependent enzymes could be modulating the beneficial effects observed in this study. These possibilities warrant future investigation.

The daily dose of intake is a critical issue for selenium therapeutics. There is no a standard of the official guideline for the use of selenium. The WHO has made recommendation on the dose of selenium for adults to be 30-40 *μ*g/day and stated that daily intake up to 400 *μ*g selenium is safe [26]. Further, there are big differences of recommended dose of selenium among countries in consideration of differences in geographical and racial natures as well as in living styles of particular populations. The optimal doses of daily selenium supplementation are 31.5-200 *μ*g in patients with metabolic diseases and 82.4-200 *μ*g for patients with metabolic disorders [27]. The daily dose of A-SeQDs in patients will be investigated by us in future.

In conclusions, this study supports that we have made a novel drug of A-SeQDs, which activate DHFR to increase BH4 salvage pathway in endothelial cells (Supplementary Figure 5). In this way, A-SeQDs delayed the progression of PAH in monocrotaline-injected mice. The findings that A-SeQDs recouple eNOS may increase the applications because endothelial dysfunction is common at the initiation and in the progress in multiple cardiovascular diseases including atherosclerosis [28, 29], hypertension [16], vascular stiffness [30], and diabetic complications [31].

## MATERIALS AND METHODS

A full description of materials and methods used in this study can be found in the Supplementary Materials.

### Materials and animals

Bovine serum albumin (BSA), Na_2_S_2_O_3_, diaminofluore scein (DAF), dihydroethidium (DHE), monocrotaline, eNOS activity assay kit, and dihydrofolate reductase (DHFR) activity kit were purchased from Sigma chemical Co, USA. Primary antibodies against DHFR and GAPDH, and secondary antibody were obtained from Santa Cruz Biotechnology. Selenium lentils were grown on naturally selenium-rich soil in Inter Monggol, China. Male C57B16 wildtype (*WT*) mice (20-25 g, 8-12 weeks old) were purchased from the Laboratory Animal Center in Henan province, China. DHFR gene deletion (*DHFR^–/–^*) mice were generated from Sai-Ye Gene Company (Guangzhou, China) as described previously [5]. The animal protocol was reviewed and approved by Henan Normal University, Institute of Animal Care and Use Committee, which conformed to the NIH Guide for the Care and Use of Laboratory Animals.

### Patients and sample processing

Eleven patients with PAH and twelve healthy persons were recruited into this study. PAH was diagnosed as the systolic PAP is over 35 mmHg. Blood was collected from human subjects. The procedures must be in accordance with the ethical standards of the responsible committee on human experimentation or with the Helsinki Declaration of 1975. The study protocol was approved by the Ethical Committee of Xinxiang Medical University, and informed written consent was given prior to the inclusion of subjects in the study.

### Preparations of A-SeQDs and C-SeQDs

As described previously [32], BSA was added into the reaction system (pH=6.0) after selenium powder was added into aqueous solution of sodium sulfite (Supplementary Figure 1A). For A-SeQDs, the reaction system was incubated at 25° C for 12 hrs. For C-SeQDs, the reaction was performed at 80° C for 36 hrs. After reaction, the dispersion was centrifuged, washed by ddH_2_O, and freeze-dried (Supplementary Figure 1B).

### Establishment of PAH model and measurement of pulmonary arterial pressure (PAP)

General anesthesia was maintained by sevoflurane inhalation (1.0-2.0%, with 100% oxygen). Body temperature was maintained by an electric heating table. Under anesthesia, mice were induced to PAH through a subcutaneous injection in a single dose of monocrotaline (100 mg/kg) as described previously [33]. PAP was monitored by implanting a radio telemetry (Dataquest A.R.T. 3.1; Data Sciences). Right ventricle systolic pressures (RVSP) were measured by a fluid-filled sensing catheter inserted into right ventricle (RV) through the jugular vein and connected to the transmitter (model TA11PA-40). The ratio of RV to left ventricle (LV) plus septum (S) weights [RV/(LV+S)] were calculated. The animals were kept in separate cages, and subcutaneous injection of antibiotics (Baytril 5%, 10–20 mg/kg) and analgesics (buprenorphine, 0.1 mL/kg) were administered after measurement.

### Measurement of biopterins by high performance liquid chromatography (HPLC)

The levels of biopterins, including BH4 and BH2, were determined as previously described with some modifications [34]. Quantifications of BH4 and BH2 were done by comparison with authentic external standards and normalized to sample protein content.

### DHFR activity measured by HPLC

To determine DHFR activity in tissues, we adapted a highly sensitive HPLC method as described previously [35].

### Western blotting

As described previously [36], lung tissues or cell lysates were homogenized on ice in cell-lysis buffer. Total proteins of 20 μg were loaded to SDS-PAGE and then transferred to membrane. Membrane was incubated with a 1:1000 dilution of primary antibody, followed by a 1:2000 dilution of horseradish peroxidase-conjugated secondary antibody. Protein bands were visualized by enhanced chemiluminescence (GE Healthcare).

### Detections of NO and ROS

As described previously [37], NO was detected using the fluorescent probe DAF, and ROS was detected using the fluorescent probe DHE.

### Measurement of eNOS activity

The activity of eNOS was monitored by L- [^3^H]-citrulline production from L- [^3^H]-arginine as described previously [38].

### Determination of selenium concentration

Samples were immediately prepared and stored at -80° C until analysis. Selenium concentrations were determined by a Spectra AA 220Z (Varian) with carbon-furnace atomic-absorption spectrometry and Zeeman compensation [39]. The sensitivity of this method, defined as the quantity giving an absorbance of 1%, was 1 μg/l of selenium. Using a dilution of 1:11, therefore the sensitivity was 11 μg/l for measurement.

### Statistical analysis

Data were reported as the mean ± S.E.M. Multiple comparisons over two groups were performed using a one-way ANOVA followed by Tukey post-hoc tests. Two-sided P-values < 0.05 were considered significant.

## Supplementary Material

Supplementary Materials and Methods

Supplementary Figures

Supplementary Tables

## References

[1] Budhiraja R, Tuder RM, Hassoun PM. Endothelial dysfunction in pulmonary hypertension. Circulation. 2004; 109:159–65. 10.1161/01.CIR.0000102381.57477.50 14734504

[2] Li P, Yin YL, Guo T, Sun XY, Ma H, Zhu ML, Zhao FR, Xu P, Chen Y, Wan GR, Jiang F, Peng QS, Liu C, et al. Inhibition of aberrant MicroRNA-133a expression in endothelial cells by statin prevents endothelial dysfunction by targeting GTP cyclohydrolase 1 in vivo. Circulation. 2016; 134:1752–65. 10.1161/CIRCULATIONAHA.116.017949 27765794 PMC5120771

[3] Khoo JP, Zhao L, Alp NJ, Bendall JK, Nicoli T, Rockett K, Wilkins MR, Channon KM. Pivotal role for endothelial tetrahydrobiopterin in pulmonary hypertension. Circulation. 2005; 111:2126–33. 10.1161/01.CIR.0000162470.26840.89 15824200

[4] Nandi M, Miller A, Stidwill R, Jacques TS, Lam AA, Haworth S, Heales S, Vallance P. Pulmonary hypertension in a GTP-cyclohydrolase 1-deficient mouse. Circulation. 2005; 111:2086–90. 10.1161/01.CIR.0000163268.32638.F4 15824199

[5] Li Q, Youn JY, Siu KL, Murugesan P, Zhang Y, Cai H. Knockout of dihydrofolate reductase in mice induces hypertension and abdominal aortic aneurysm via mitochondrial dysfunction. Redox Biol. 2019; 24:101185. 10.1016/j.redox.2019.101185 30954686 PMC6451172

[6] Dikalova A, Aschner JL, Kaplowitz MR, Summar M, Fike CD. Tetrahydrobiopterin oral therapy recouples eNOS and ameliorates chronic hypoxia-induced pulmonary hypertension in newborn pigs. Am J Physiol Lung Cell Mol Physiol. 2016; 311:L743–53. 10.1152/ajplung.00238.2016 27542807 PMC5142125

[7] Liu H, Xu H, Huang K. Selenium in the prevention of atherosclerosis and its underlying mechanisms. Metallomics. 2017; 9:21–37. 10.1039/c6mt00195e 28009916

[8] Pilarczyk B, Tomza-Marciniak A, Pilarczyk R, Hendzel D, Błaszczyk B, Bąkowska M. Tissue distribution of selenium and effect of season and age on selenium content in roe deer from northwestern Poland. Biol Trace Elem Res. 2011; 140:299–307. 10.1007/s12011-010-8705-2 20446055

[9] Kim HY, Picciano MF, Wallig MA, Milner JA. The role of selenium nutrition in the development of neonatal rat lung. Pediatr Res. 1991; 29:440–45. 10.1203/00006450-199105010-00006 1896247

[10] Gerhardsson L, Brune D, Nordberg GF, Wester PO. Selenium and other trace elements in lung tissue in smelter workers relationship to the occurrence of lung cancer. Acta Pharmacol Toxicol (Copenh). 1986 (Suppl 7); 59:256–59. 10.1111/j.1600-0773.1986.tb02756.x 3776574

[11] Stanwix H. Interview: from traditional polymer science to nanomedicine: the interplay between disciplines to drive innovation. Nanomedicine (Lond). 2012; 7:1125–28. 10.2217/nnm.12.89 22931446

[12] Liu W, Li X, Wong YS, Zheng W, Zhang Y, Cao W, Chen T. Selenium nanoparticles as a carrier of 5-fluorouracil to achieve anticancer synergism. ACS Nano. 2012; 6:6578–91. 10.1021/nn202452c 22823110

[13] Zamani Moghaddam AK, Mehraei Hamzekolaei MH, Khajali F, Hassanpour H. Role of selenium from different sources in prevention of pulmonary arterial hypertension syndrome in broiler chickens. Biol Trace Elem Res. 2017; 180:164–70. 10.1007/s12011-017-0993-3 28317078

[14] Ryan JJ, Archer SL. The right ventricle in pulmonary arterial hypertension: disorders of metabolism, angiogenesis and adrenergic signaling in right ventricular failure. Circ Res. 2014; 115:176–88. 10.1161/CIRCRESAHA.113.301129 24951766 PMC4112290

[15] Schermuly RT, Ghofrani HA, Wilkins MR, Grimminger F. Mechanisms of disease: pulmonary arterial hypertension. Nat Rev Cardiol. 2011; 8:443–55. 10.1038/nrcardio.2011.87 21691314 PMC7097518

[16] Wang S, Xu J, Song P, Wu Y, Zhang J, Chul Choi H, Zou MH. Acute inhibition of guanosine triphosphate cyclohydrolase 1 uncouples endothelial nitric oxide synthase and elevates blood pressure. Hypertension. 2008; 52:484–90. 10.1161/hypertensionaha.108.112094 18645049 PMC3523107

[17] Krohn RM, Lemaire M, Negro Silva LF, Lemarié C, Bolt A, Mann KK, Smits JE. High-selenium lentil diet protects against arsenic-induced atherosclerosis in a mouse model. J Nutr Biochem. 2016; 27:9–15. 10.1016/j.jnutbio.2015.07.003 26500064

[18] Mehta U, Kang BP, Bansal G, Bansal MP. Studies of apoptosis and bcl-2 in experimental atherosclerosis in rabbit and influence of selenium supplementation. Gen Physiol Biophys. 2002; 21:15–29. 12168721

[19] Omrani H, Golmohamadi S, Pasdar Y, Jasemi K, Almasi A. Effect of selenium supplementation on lipid profile in hemodialysis patients. J Renal Inj Prev. 2016; 5:179–82. 10.15171/jrip.2016.38 27689119 PMC5039985

[20] Weekley CM, Harris HH. Which form is that? The importance of selenium speciation and metabolism in the prevention and treatment of disease. Chem Soc Rev. 2013; 42:8870–94. 10.1039/c3cs60272a 24030774

[21] Wang S, Zhang M, Liang B, Xu J, Xie Z, Liu C, Viollet B, Yan D, Zou MH. AMPKalpha2 deletion causes aberrant expression and activation of NAD(P)H oxidase and consequent endothelial dysfunction in vivo: role of 26S proteasomes. Circ Res. 2010; 106:1117–28. 10.1161/CIRCRESAHA.109.212530 20167927 PMC2920052

[22] Zhou SN, Lu JX, Wang XQ, Shan MR, Miao Z, Pan GP, Jian X, Li P, Ping S, Pang XY, Bai YP, Liu C, Wang SX. S-nitrosylation of prostacyclin synthase instigates nitrate cross-tolerance in vivo. Clin Pharmacol Ther. 2019; 105:201–09. 10.1002/cpt.1094 29672839

[23] Liang WJ, Zhou SN, Shan MR, Wang XQ, Zhang M, Chen Y, Zhang Y, Wang SX, Guo T. AMPKα inactivation destabilizes atherosclerotic plaque in streptozotocin-induced diabetic mice through AP-2α/miRNA-124 axis. J Mol Med (Berl). 2018; 96:403–12. 10.1007/s00109-018-1627-8 29502204

[24] Zhu ML, Wang G, Wang H, Guo YM, Song P, Xu J, Li P, Wang S, Yang L. Amorphous nano-selenium quantum dots improve endothelial dysfunction in rats and prevent atherosclerosis in mice through Na^+^/H^+^ exchanger 1 inhibition. Vascul Pharmacol. 2019; 115:26–32. 10.1016/j.vph.2019.01.005 30695730

[25] Zhang HM, Liu MY, Lu JX, Zhu ML, Jin Q, Ping S, Li P, Jian X, Han YL, Wang SX, Li XY. Intracellular acidosis via activation of Akt-Girdin signaling promotes post ischemic angiogenesis during hyperglycemia. Int J Cardiol. 2019; 277:205–11. 10.1016/j.ijcard.2018.08.028 30316647

[26] Kieliszek M, Błażejak S. Current knowledge on the importance of selenium in food for living organisms: a review. Molecules. 2016; 21:609. 10.3390/molecules21050609 27171069 PMC6274134

[27] Wang N, Tan HY, Li S, Xu Y, Guo W, Feng Y. Supplementation of micronutrient selenium in metabolic diseases: its role as an antioxidant. Oxid Med Cell Longev. 2017; 2017:7478523. 10.1155/2017/7478523 29441149 PMC5758946

[28] Antoniades C, Shirodaria C, Warrick N, Cai S, de Bono J, Lee J, Leeson P, Neubauer S, Ratnatunga C, Pillai R, Refsum H, Channon KM. 5-methyltetrahydrofolate rapidly improves endothelial function and decreases superoxide production in human vessels: effects on vascular tetrahydrobiopterin availability and endothelial nitric oxide synthase coupling. Circulation. 2006; 114:1193–201. 10.1161/CIRCULATIONAHA.106.612325 16940192

[29] Kawashima S, Yokoyama M. Dysfunction of endothelial nitric oxide synthase and atherosclerosis. Arterioscler Thromb Vasc Biol. 2004; 24:998–1005. 10.1161/01.ATV.0000125114.88079.96 15001455

[30] Zhu ML, Sun RL, Zhang HY, Zhao FR, Pan GP, Zhang C, Song P, Li P, Xu J, Wang S, Yin YL. Angiotensin II type 1 receptor blockers prevent aortic arterial stiffness in elderly patients with hypertension. Clin Exp Hypertens. 2019; 41:657–61. 10.1080/10641963.2018.1529781 30311805

[31] Alp NJ, Mussa S, Khoo J, Cai S, Guzik T, Jefferson A, Goh N, Rockett KA, Channon KM. Tetrahydrobiopterin-dependent preservation of nitric oxide-mediated endothelial function in diabetes by targeted transgenic GTP-cyclohydrolase I overexpression. J Clin Invest. 2003; 112:725–35. 10.1172/JCI17786 12952921 PMC182196

[32] Wang G, Guo Y, Yang G, Yang L, Ma X, Wang K, Zhu L, Sun J, Wang X, Zhang H. Mitochondria-mediated protein regulation mechanism of polymorphs-dependent inhibition of nanoselenium on cancer cells. Sci Rep. 2016; 6:31427. 10.1038/srep31427 27514819 PMC4981849

[33] Zhu TT, Zhang WF, Yin YL, Liu YH, Song P, Xu J, Zhang MX, Li P. MicroRNA-140-5p targeting tumor necrosis factor-α prevents pulmonary arterial hypertension. J Cell Physiol. 2019; 234:9535–50. 10.1002/jcp.27642 30367500

[34] Fukushima T, Nixon JC. Analysis of reduced forms of biopterin in biological tissues and fluids. Anal Biochem. 1980; 102:176–88. 10.1016/0003-2697(80)90336-x 7356152

[35] Crabtree MJ, Hale AB, Channon KM. Dihydrofolate reductase protects endothelial nitric oxide synthase from uncoupling in tetrahydrobiopterin deficiency. Free Radic Biol Med. 2011; 50:1639–46. 10.1016/j.freeradbiomed.2011.03.010 21402147 PMC3121954

[36] Wang S, Zhang C, Zhang M, Liang B, Zhu H, Lee J, Viollet B, Xia L, Zhang Y, Zou MH. Activation of AMP-activated protein kinase α2 by nicotine instigates formation of abdominal aortic aneurysms in mice in vivo. Nat Med. 2012; 18:902–10. 10.1038/nm.2711 22561688 PMC3559018

[37] Thomas S, Kotamraju S, Zielonka J, Harder DR, Kalyanaraman B. Hydrogen peroxide induces nitric oxide and proteosome activity in endothelial cells: a bell-shaped signaling response. Free Radic Biol Med. 2007; 42:1049–61. 10.1016/j.freeradbiomed.2007.01.005 17349932 PMC2692187

[38] Wang S, Peng Q, Zhang J, Liu L. Na+/H+ exchanger is required for hyperglycaemia-induced endothelial dysfunction via calcium-dependent calpain. Cardiovasc Res. 2008; 80:255–62. 10.1093/cvr/cvn179 18591204

[39] Lubos E, Sinning CR, Schnabel RB, Wild PS, Zeller T, Rupprecht HJ, Bickel C, Lackner KJ, Peetz D, Loscalzo J, Münzel T, Blankenberg S. Serum selenium and prognosis in cardiovascular disease: results from the AtheroGene study. Atherosclerosis. 2010; 209:271–77. 10.1016/j.atherosclerosis.2009.09.008 19836749 PMC2921799

