# Supplementary material for: Amorphous nano-selenium quantum dots prevent pulmonary arterial hypertension through recoupling endothelial nitric oxide synthase
Source: Aging (Albany NY). 2020 Dec 15;13(3):3368–85. doi: 10.18632/aging.202215 (PMC7906187; doi:10.18632/aging.202215)
Supplement: Supplementary Materials and Methods [file aging-13-202215-s001.pdf]

SUPPLEMENTARY MATERIALS AND METHODS

Preparation of SeQDs

As we described previously [1, 2], selenium powder was added into aqueous solution of sodium sulfite (50 mM) at 95° C. Then bovine serum albumin (70 mg) was added into the reaction system and the pH value was adjusted to 6.0. The reaction system instantly changed to red color (Supplementary Figure 1A). Subsequently, the reaction system was incubated at 20° C for 12 h. Finally, the dispersion was centrifuged, washed, and freeze-dried (Supplementary Figure 1B). The procedures of C-SeQDs preparations were similar to A-SeQDs, except that the reaction system was incubated at 80° C for 24 h. The size and morphology of two SeQDs were characterized by HR-TEM (JEOL JEM-2100) with the acceleration voltage of 200 KV. The crystal phases were determined by XRD using a D8ADVANCE X-ray diffractometer (Bruker axs Com.) with graphite monochromatized Cu Kα radiation (λ = 0.15406 nm) in the 2θ range of 20-80°. EDX spectrum was recorded on a GENESIS system (EDAX Inc.) attached to the JEM-2100 microscope. The photoluminescence (PL) measurements were carried out on a HITACHI FP-6500 spectrophotometer. ζ potential was measured on a Nano-ZS instrument in triplicate in H_2O, PBS, or DMEM.

Materials and animals

Bovine serum albumin (BSA), $Na_2S_2O_3$, diaminofluorescein (DAF), dihydroethidium (DHE), monocrotaline, eNOS activity assay kit, and dihydrofolate reductase (DHFR) activity kit were purchased from Sigma chemical Co, USA. Primary antibody against DHFR and GAPDH, and secondary antibody were obtained from Santa Cruz Biotechnology. Selenium lentils were grown on naturally selenium-rich soil in Inter Monggol, China. C57B16 wildtype (*WT*) mice (20-25 g) were purchased from the Laboratory Animal Center in Henan province, China. DHFR gene deletion ($DHFR^{-/-}$) mice were generated from Ji-Kai Gene Company (Guangzhou, China) as described previously [3]. They were housed in a temperature-controlled environment (21±1° C) and humidity (40-60%) with a 12 h light/dark cycle, and were provided free access to tap water. This animal study was carried out in strictly accordance with the recommendations in the Guide for the Care and Use of Laboratory Animals of the National Institutes of Health. The animal protocol was reviewed and approved by Henan Normal University, Institute of Animal Care and Use Committee.

Establishment of PAH model

General anesthesia was maintained by sevoflurane inhalation (1.0-2.0%, with 100% oxygen). Body temperature was maintained by an electric heating table. Under anaesthesia, mice were induced to PAH through a subcutaneous injection in a single dose of monocrotaline (100 mg/kg) based on the previous study [4]. Pulmonary arterial pressure (PAP) was monitored by implanting a radio telemetry (Dataquest A.R.T. 3.1; Data Sciences). RV pressures were measured by a fluid-filled sensing catheter inserted into the RV through the jugular vein and connected to the transmitter (model TA11PA-40), which sends the signal to a remote receiver (model RPC-1) and data exchanger connected to a computer. The pressure wave form was monitored online at 30-minute intervals. The animals were kept in separate cages, and subcutaneous injection of antibiotics (Baytril 5%, 10–20 mg/kg) and analgesics (buprenorphine, 0.1 mL/kg) were administered after surgery.

Protocols of animal studies

As described in Supplementary Figure 3A, mice (C57B16 and $DHFR^{-/-}$) were randomly classified into each group. Mice were pretreated with organic selenium, A-SeQDs, and C-SeQDs for 4 weeks. The dose of selenium element was 30 mg/kg/day in organic selenium, A-SeQDs, and C-SeQDs. The PAH model was induced by a single injection of monocrotaline for 3 weeks. Regular diet containing selenium lentils were produced by Te-Luo-Fei Company (Nantong, China). Other than selenium content, ingredients of all diets were the same as in commercial rodent chow, ensuring that all other nutrients met or exceeded the requirements for standard rodent nutrition.

Histological examination

After experiments, animals were then killed and tissues were collected, snap frozen, and stored at −80° C for biochemical measurements. Hearts were dissected and weighed, and the ratio of right ventricle (RV) to left ventricle plus the septum mass was used as an index of right ventricular hypertrophy. The left lung and RV were fixed with 10% formalin in phosphate-buffered saline and processed for elastic Van Gieson and hematoxylin and eosin (HE) staining. Vessels less than 100 μm in peripheral lung were counted blindly under microscope, and pulmonary vascular remodeling was expressed as the proportion of vessels with double elastic lamina (>50%) to total vessels counted (percentage total muscularized vessels). Axio-Vision software (Carl Zeiss) was used to quantifying RV

cardiomyocyte cross section area and myocyte diameter from transversely cut cardiomyocytes using H&E sections.

Measurement of Biopterins

The levels of BH4 and total biopterins were determined as previously described with some modification [5–7]. Briefly, homogenates of aorta or cell lysates were suspended in distilled water containing 5 mM dithioerythrol, centrifuged at 12000g at 4° C for 10 min, and then subjected to oxidation in acid or base. To 100 µl aliquot of supernatant, 20 µl of 0.5 M HCl and 0.05 M iodine were added for acidic oxidation, and 20 µl of 0.5 M NaOH plus 0.05 M iodine were added for basic oxidation. After incubation for 1h in the dark at room temperature, 20 µl HCl was added to the basic oxidation only. All mixtures received 20 µl 0.1 M ascorbic acid for the reduction of excess iodine. Samples were then centrifuged for 10 min at 12000g at 4° C. Biopterin concentrations were determined by HPLC with a PR-C18 column. Elution was at a rate of 1.0 ml/min of 50 mM potassium phosphate buffer, pH 3.0. Fluorescence was detected with an excitation at 350 nm and emission at 440 nm. Quantifications of BH4 and BH2 were done by comparison with authentic external standards and normalized to sample protein content. Total biopterin levels are expressed as the sum of detectable BH4 and BH2.

Measurement of DHFR activity by HPLC

To determine DHFR activity in tissues, we adapted a highly sensitive HPLC method as described previously [8]. Briefly, tissue homogenates were incubated with dihydrofolate (50 µM) for 20 min at 37° C, in a 0.1 M potassium phosphate assay buffer (pH 7.4) containing 200 µM NADPH, 1 mM dithiothreitol (DTT), 0.5 mM KCl, 1 mM EDTA, and 20 mM sodium ascorbate. After 30 min at 37° C, the reaction was terminated by the addition of 0.2 M trichloroacetic acid. A stabilization solution (200 mg of sodium ascorbate and 30 mg of DTT in 1 ml of water) was then added and samples were stored at −20° C until analysis. The accumulation of the reaction products, tetrahydrofolate (THF) and methyltetrahydrofolate (MeTHF), was then quantified by HPLC using fluorescence detection (295 nm for excitation and 365 nm for emission). Dihydrofolate (DHF) was detectable only at concentrations over 1000 times more than those of both THF and MeTHF.

Western blotting

As described previously [9], aortic tissues were homogenized on ice in cell-lysis buffer (20 mM Tris-HCl, pH 7.5, 150 mM NaCl, 1 mM Na_2EDTA, 1 mM EGTA, 1% Triton, 2.5 mM sodium pyrophosphate, 1 mM beta-glycerophosphate, 1 mM Na_3VO_4, 1 µg/ml leupeptin) and 1 mM PMSF. Cell was lysed with cell-lysis buffer. The protein content was assayed by BCA protein assay reagent (Pierce, USA). 20 µg proteins were loaded to SDS-PAGE and then transferred to membrane. Membrane was incubated with a 1:1000 dilution of primary antibody, followed by a 1:2000 dilution of horseradish peroxidase- conjugated secondary antibody. Protein bands were visualized by ECL (GE Healthcare). The intensity (area X density) of the individual bands on Western blots was measured by densitometry (model GS-700, Imaging Densitometer; Bio-Rad). The background was subtracted from the calculated area. We used control as 100%.

Detection of NO

NO productions in tissues were detected using the fluorescent probe DAF as described previously [10]. Briefly, fresh tissue isolated from mice were incubated with 10 µM DAF for 30 min at 37° C immediately after isolation, then washing with PBS twice. The DAF fluorescent intensity was recorded by fluorescent reader at the wave of excitation (485 nm) and emission (545 nm). Control was setup as 100%.

Detection of ROS

ROS productions in tissues were detected using the fluorescent probe DHE as described previously. [11] Briefly, fresh tissue isolated from mice were incubated with 10 µM DHE for 30 min at 37° C immediately after isolation, then washing with PBS twice. The DHE fluorescent intensity in cells was recorded by fluorescent reader at the wave of excitation (485 nm) and emission (645 nm). The DHE fluorescence intensity in homogenates of aorta was assayed by HPLC according to the method we used before. Control was setup as 100%.

eNOS activity assay

eNOS activity was monitored by L-[^{3}H]-citrulline production from L-[^{3}H]-arginine as described previously [12]. Briefly, protein samples were incubated in reaction buffer containing 1 mM L-arginine, 100 mM NADPH, 1 mM tetrahydrobiopterin, 0.2 µCi of L-[^{3}H]-arginine (>66 Ci/mmol), and Nω-hydroxy-nor-L-arginine (10 µM). The reaction was performed at 37° C for 15 min and the mixture was separated by Dowex-50W ion-exchange chromatography in 20 mM HEPES (pH 5.5), 2 mM EDTA, and 2 mM EGTA, and the flow-through was used for liquid scintillation counting.

Patients and sample processing

Eleven patients with PAH and twelve healthy persons were recruited into this study. The demographic data were presented in Supplementary Table 2. PAH was diagnosed as the systolic PAP is over 35 mmHg. Bloods were collected from human subjects to measure biopterins including BH4 and BH2, and DHFR activity in white blood cells. Serum NO level was also determined by Griess method. The procedures must be in accordance with the ethical standards of the responsible committee on human experimentation or with the Helsinki Declaration of 1975. The study protocol was approved by the Ethical Committee of Xinxiang Medical University, and informed consent was obtained from the human subjects.

Statistical analysis

Data are reported as the mean ± S.E.M. Multiple comparisons over two groups were performed using a one-way ANOVA followed by Tukey post-hoc tests or Bonferroni post-hoc analyses. Comparison between two groups was performed using student's t test. Two-sided P-values < 0.05 were considered significant.

REFERENCES

1. Zhu ML, Wang G, Wang H, Guo YM, Song P, Xu J, Li P, Wang S, Yang L. Amorphous nano-selenium quantum dots improve endothelial dysfunction in rats and prevent atherosclerosis in mice through Na^+/H^+ exchanger 1 inhibition. Vascul Pharmacol. 2019; 115:26–32. https://doi.org/10.1016/j.vph.2019.01.005 PMID:30695730

2. Wang G, Guo Y, Yang G, Yang L, Ma X, Wang K, Zhu L, Sun J, Wang X, Zhang H. Mitochondria-mediated protein regulation mechanism of polymorphs-dependent inhibition of nanoselenium on cancer cells. Sci Rep. 2016; 6:31427. https://doi.org/10.1038/srep31427 PMID:27514819

3. Li Q, Youn JY, Siu KL, Murugesan P, Zhang Y, Cai H. Knockout of dihydrofolate reductase in mice induces hypertension and abdominal aortic aneurysm via mitochondrial dysfunction. Redox Biol. 2019; 24:101185. https://doi.org/10.1016/j.redox.2019.101185 PMID:30954686

4. Zhu TT, Zhang WF, Yin YL, Liu YH, Song P, Xu J, Zhang MX, Li P. MicroRNA-140-5p targeting tumor necrosis factor-α prevents pulmonary arterial hypertension. J Cell Physiol. 2019; 234:9535–50. https://doi.org/10.1002/jcp.27642 PMID:30367500

5. Fukushima T, Nixon JC. Analysis of reduced forms of biopterin in biological tissues and fluids. Anal Biochem. 1980; 102:176–88. https://doi.org/10.1016/0003-2697(80)90336-x PMID:7356152

6. Wang S, Xu J, Song P, Viollet B, Zou MH. In vivo activation of AMP-activated protein kinase attenuates diabetes-enhanced degradation of GTP cyclohydrolase I. Diabetes. 2009; 58:1893–901. https://doi.org/10.2337/db09-0267 PMID:19528375

7. Wang S, Xu J, Song P, Wu Y, Zhang J, Chul Choi H, Zou MH. Acute inhibition of guanosine triphosphate cyclohydrolase 1 uncouples endothelial nitric oxide synthase and elevates blood pressure. Hypertension. 2008; 52:484–90. https://doi.org/10.1161/HYPERTENSIONAHA.108.112094 PMID:18645049

8. Crabtree MJ, Hale AB, Channon KM. Dihydrofolate reductase protects endothelial nitric oxide synthase from uncoupling in tetrahydrobiopterin deficiency. Free Radic Biol Med. 2011; 50:1639–46. https://doi.org/10.1016/j.freeradbiomed.2011.03.010 PMID:21402147

9. Wang S, Zhang C, Zhang M, Liang B, Zhu H, Lee J, Viollet B, Xia L, Zhang Y, Zou MH. Activation of AMP-activated protein kinase α2 by nicotine instigates formation of abdominal aortic aneurysms in mice in vivo. Nat Med. 2012; 18:902–10. https://doi.org/10.1038/nm.2711 PMID:22561688

10. Thomas S, Kotamraju S, Zielonka J, Harder DR, Kalyanaraman B. Hydrogen peroxide induces nitric oxide and proteosome activity in endothelial cells: a bell-shaped signaling response. Free Radic Biol Med. 2007; 42:1049–61. https://doi.org/10.1016/j.freeradbiomed.2007.01.005 PMID:17349932

11. Xu J, Xie Z, Reece R, Pimental D, Zou MH. Uncoupling of endothelial nitric oxidase synthase by hypochlorous acid: role of NAD(P)H oxidase-derived superoxide and peroxynitrite. Arterioscler Thromb Vasc Biol. 2006; 26:2688–95. https://doi.org/10.1161/01.ATV.0000249394.94588.82 PMID:17023679

12. Wang S, Peng Q, Zhang J, Liu L. Na+/H+ exchanger is required for hyperglycaemia-induced endothelial dysfunction via calcium-dependent calpain. Cardiovasc Res. 2008; 80:255–62. https://doi.org/10.1093/cvr/cvn179 PMID:18591204
