# Supplementary material for: Amorphous nano-selenium quantum dots prevent pulmonary arterial hypertension through recoupling endothelial nitric oxide synthase
Source: Aging (Albany NY). 2020 Dec 15;13(3):3368–85. doi: 10.18632/aging.202215 (PMC7906187; doi:10.18632/aging.202215)
Supplement: Supplementary Figures [file aging-13-202215-s002.pdf]

SUPPLEMENTARY FIGURES

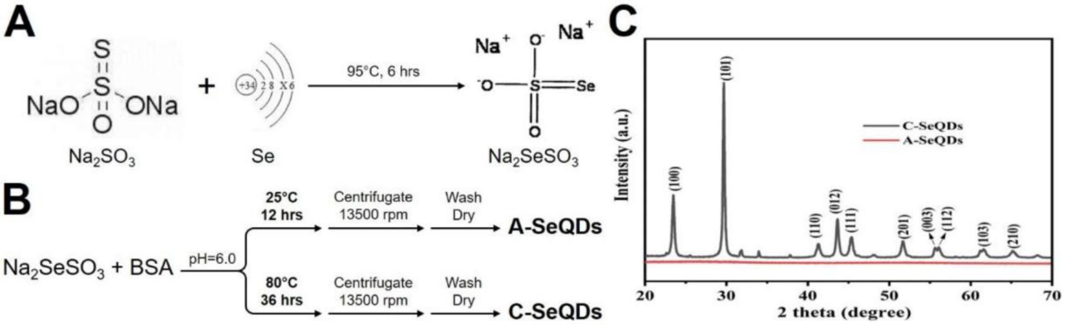

Supplementary Figure 1. The steps of SeQDs preparations. (**A**) The mixtures of $Na_2S_2O_3$ and selenium were heated at 95° C for 6 hours to get Na_2SeSO_3. (**B**) Na_2SeSO_3 was treated with bovine serum albumin (BSA) under 25° C for 12 hours or under 80° C for 36 hours at pH=6.0. Then the reaction mixture was centrifuged at 13500 rpm, followed by wash with ddH_2O. Finally, let the mixture dry at room temperature. (**C**) The diffraction peaks of A-SeQDs and C-SeQDs were assayed by X-ray diffraction pattern.

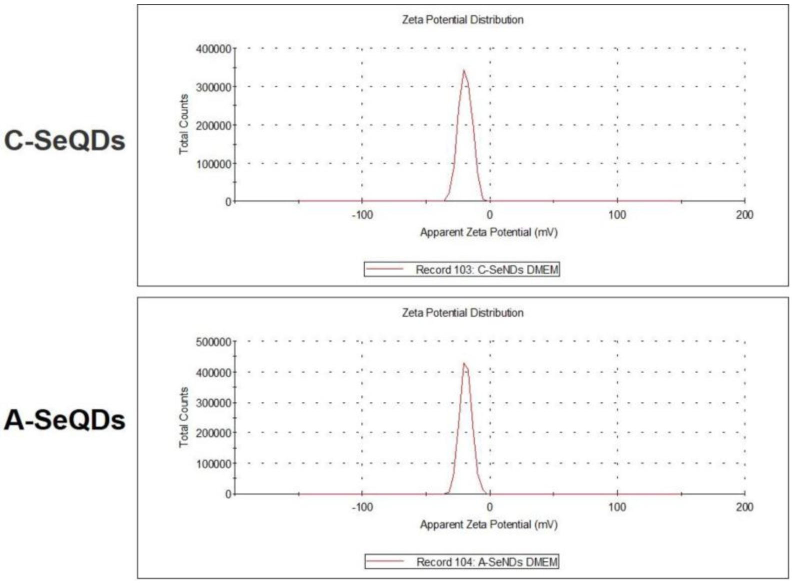

Supplementary Figure 2. The representative traces of ξ-potential in DMEM. The stabilities of A-SeQDs and C-SeQDs in DMEM were examined by performing ξ-potential measurement and the representative traces were shown.

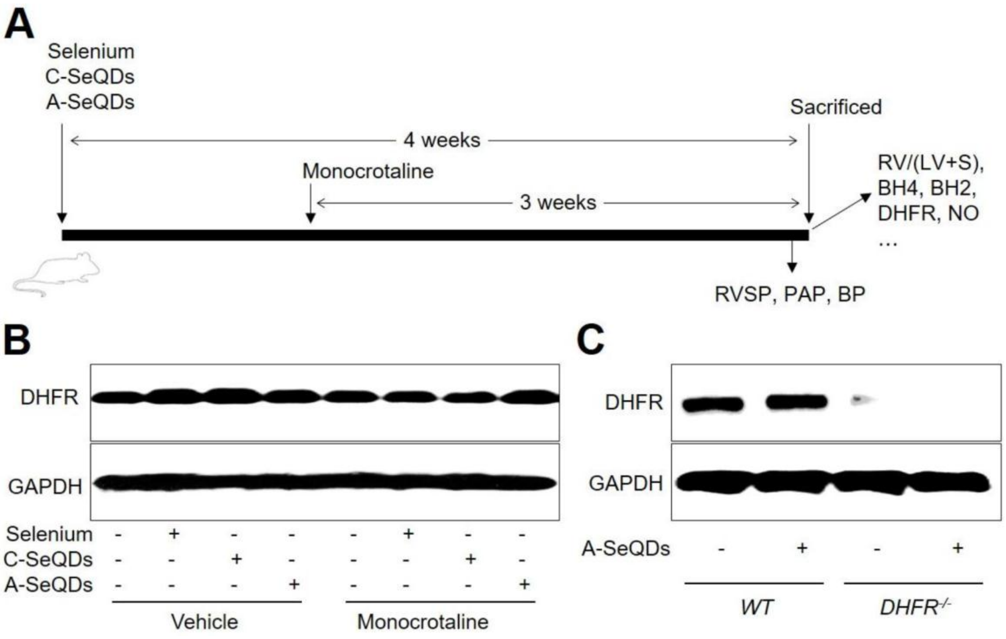

Supplementary Figure 3. The protocols of animal experiments. (**A**) Mice were given treatments of selenium, C-SeQDs, A-SeQDs in regular diet one week prior to a single intraperitoneal injection of 100 mg/kg monocrotaline or vehicle. Three weeks after injection, right ventricle systolic pressure (RVSP), mean pulmonary arterial pressure (PAP), systemic mean blood pressure, and the ratio of right ventricle (RV) to left ventricle (LV) plus septum (S) weights [RV/(LV+S)] were measured. (**B**) Pulmonary artery isolated from mice treated with selenium, C-SeQDs, and A-SeQDs were subjected to measure DHFR protein by Western blots. (**C**) Pulmonary artery isolated from *WT* and *DHFR*$^{-/-}$ mice were subjected to measure DHFR protein by Western blots. 10-15 mice were in each group.

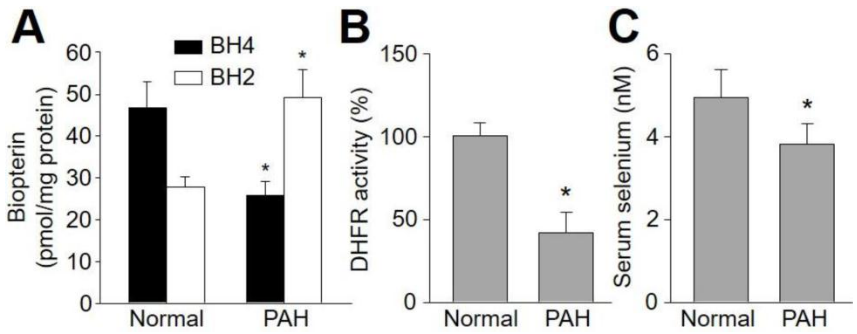

Supplementary Figure 4. Decreased BH4 and DHFR activity in human patients with PAH. The demographic data were presented in Supplementary Table 2. Bloods were collected from human patients and subjected to measure biopterins including BH4 and BH2 in (**A**), DHFR activity in (**B**) and serum NO levels in (**C**). All data were expressed as mean ± SEM. **P*<0.05 *VS* Normal. A student's t test was used to produce the P values.

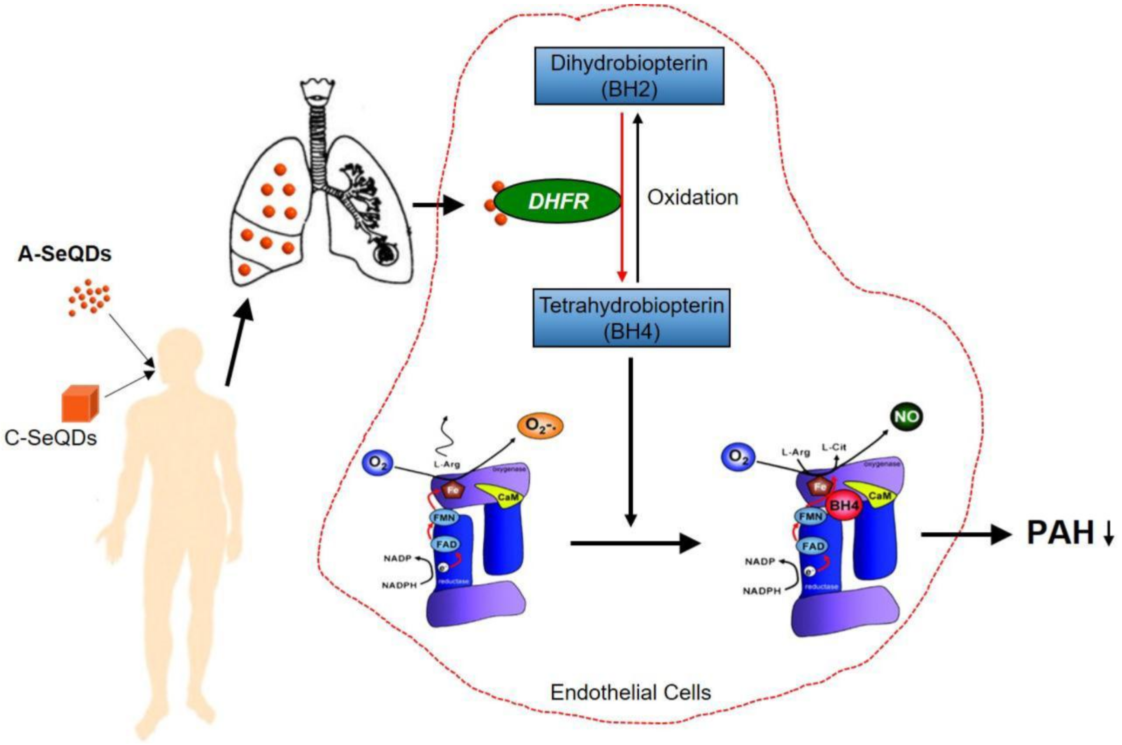

Supplementary Figure 5. Proposed mechanisms of A-SeQDs treating PAH. Both C-SeQDs and A-SeQDs are able to be absorbed in body, while, A-SeQDs are distributed into lung. In pulmonary artery endothelial cells, A-SeQDs activate DHFR in increase BH4 salvage pathway to upregulate endogenous BH4 biosynthesis. BH4 recouples eNOS to produce NO but not ROS. NO induces the relaxation of vascular smooth muscle cell through cGMP-dependent signaling. In this way, A-SeQDs prevent endothelial dysfunction of pulmonary artery and alleviate PAH.
