# Supplementary material for: Amorphous nano-selenium quantum dots prevent pulmonary arterial hypertension through recoupling endothelial nitric oxide synthase
Source: Aging (Albany NY). 2020 Dec 15;13(3):3368–85. doi: 10.18632/aging.202215 (PMC7906187; doi:10.18632/aging.202215)
Supplement: Supplementary Tables [file aging-13-202215-s003.pdf]

SUPPLEMENTARY TABLES

Supplementary Table 1. Zeta potential of C-SeQDs and A-SeQDs in different solutions.

Solutions	DMEM	PBS	ddH_2O
A-SeQDs	-18.8mV	-21.4mV	-31.4mV
C-SeQDs	-19.3mV	-20.2mV	-30.3mV

Supplementary Table 2. Demographic data for individuals with or without PAH.

ID	Gender	Ages (years old)	PAH
1	Female	78	-
2	Male	66	+
3	Male	79	-
4	Female	77	-
5	Male	85	+
6	Female	87	+
7	Male	61	+
8	Female	79	-
9	Male	83	-
10	Male	68	-
11	Male	78	+
12	Male	79	-
13	Female	83	+
14	Female	88	+
15	Male	84	-
16	Female	78	+
17	Male	76	+
18	Female	75	-
19	Male	69	+
20	Male	66	-
21	Female	72	-
22	Male	68	+
23	Female	63	-
